# Virulence of fowl adenovirus (FAdV) serotype 4 strains impacts cell proliferation and immune response of primary chicken-embryo intestinal epithelial cells

**DOI:** 10.1186/s13567-025-01541-9

**Published:** 2025-05-27

**Authors:** Katharina Kau-Strebinger, Ursula Reichart, Taniya Mitra, Beatrice Grafl, Michael Hess, Dieter Liebhart

**Affiliations:** 1 Clinical Centre for Population Medicine in Fish, Pig and Poultry, Clinical Department for Farm Animals and Food System Science, University of Veterinary Medicine Vienna, Veterinärplatz 1, 1210 Vienna, Austria; 2 Vetcore Facility for Research, University of Veterinary Medicine Vienna, Veterinärplatz 1, 1210 Vienna, Austria; 3 Present Address: Altos Labs, San Diego, USA

**Keywords:** Intestinal epithelial cells (IEC), fowl adenovirus serotype 4 (FAdV-4), viral infection dynamics, immune response, Toll-like receptors (TLR), cytokine expression, automated imaging analyses

## Abstract

Fowl adenovirus serotype 4 (FAdV-4) causes hepatitis-hydropericardium syndrome (HHS) in chickens, leading to substantial economic losses. Following oral uptake, the virus infects intestinal epithelial cells (IEC) to overcome the first entrance barrier. The initial cellular interactions and intestinal immune responses are not well understood. This study uses a primary IEC culture model to investigate infection dynamics of virulent (AG234) and non-pathogenic (KR5) FAdV-4 strains and cellular defence mechanisms. Cell growth and viral propagation were assessed at 4, 12, 24, and 48 hours post-infection (hpi) using immunofluorescence and automated image analysis. The innate immune response was assessed by the mRNA expression of the Toll-like receptors (TLR1B, TLR2B, TLR3, TLR4, and TLR21) and the cytokines (IL-1β, IL-6, IL-10, IL-13, and IFN-γ). KR5 did not significantly reduce IEC growth; notable proliferation between 4 and 48 hpi was observed. Although IEC growth was initially similar, AG234 decreased cell numbers at 48 hpi. Compared to KR5, the abundance of AG234-infected cells was already higher at 4 hpi. Nevertheless, at 48 hpi, the number of IEC infected with the virulent strain was less than KR5, albeit without significance. The AG234 infection primarily activated the immune response at 48 hpi, characterised by a significant mRNA up-regulation of TLR3, TLR21, IL-1β and INF-γ compared to the negative control. KR5 induced a substantially higher expression of IL-13 mRNA compared to the control at 48 hpi. The results show that FAdV virulence significantly affects cell growth, viral augmentation, and the immune response. The chicken IEC culture system presented in this study effectively propagates FAdVs to examine the initial stage of intestinal infection.

**Supplementary Information:**

The online version contains supplementary material available at 10.1186/s13567-025-01541-9.

## Introduction

The hepatitis-hydropericardium syndrome (HHS) of chickens was first documented in Pakistan in 1987 [1]. Later, experimental data confirmed that serotype FAdV-4, species FAdV-C, is the primary cause of HHS [2, 3]. Schachner et al. reviewed the disease’s global impact, which includes significant economic losses and high mortality [4]. It primarily affects commercial broiler flocks aged 3–7 weeks and is characterised by a pericardial sac filled with straw-coloured fluid and enlarged, friable livers [5, 6].

Histopathology findings reveal cellular degeneration and necrosis, lymphoid infiltration, and inclusion bodies predominantly in the liver. Additionally, inflammatory and degenerative processes are observed in the heart, kidneys, lungs, and intestine, as previously summarised [4]. Furthermore, highly pathogenic FAdV-4 strains lead to severe depletion of lymphocytes in the bursa of Fabricius, thymus, and spleen [7, 8]. In contrast, some other FAdV-4 strains, including KR5, were shown to be non-pathogenic [9]. Recently, the major capsid protein hexon has been reported as a virulence factor; however, other genes may also contribute, and hexon sequences of some FAdV-4 are identical despite varying virulence [10, 11].

The chicken immune system recognises pathogens such as FAdVs through pattern recognition receptors (PRRs), including Toll-like receptors (TLRs). Activated TLRs trigger signalling pathways that express cytokines, chemokines, and interferons [12]. Previous studies have examined alterations in TLR expression across various organs (liver, spleen, and bursa of Fabricius) and the different infection routes (intraocular, intramuscular, and oral) in chickens infected with FAdV-4 [13, 14]. In particular, studies on changes in the expression of various cytokines (IL-1β, IL-6, IL-8, IL-10, IL-18, TNF-α) included organs such as the heart, liver, spleen, cecal tonsil, bursa of Fabricius, thymus, and the leghorn male hepatoma (LMH) cell line [15]. Such findings indicate that the immune system responds to FAdV-4 infection by activating TLRs and producing inflammatory cytokines to antagonise the pathogen.

Notably, previous studies excluded intestinal epithelial cells (IEC), even though primary entry occurs during natural faecal oral FAdV infection [16]. In general, IEC, which line the intestinal walls, play a crucial role in digestion and immune response by forming a physicochemical barrier, facilitating nutrient absorption, and secreting immune mediators to repel pathogens [17]. The structure and function of chicken intestinal cells are similar to those of mammals; however, there are key differences, such as proliferating cells along the intestinal villi, suggesting unique cell production dynamics [18]. Furthermore, studies on the existence of Paneth cells capable of secreting antimicrobial compounds are contradictory [19, 20]. These disparities in the structure of the mammalian and avian intestine should be considered in host responses against pathogens.

Cell culture techniques have advanced significantly in biomedical research, facilitating the development of ex vivo and in vitro models for studying infection dynamics, immune responses, medication effects, and more. In this context, primary cell cultures have proven highly suitable for accurately mimicking the in vivo state [21]. However, only a single study in chickens describes the use of primary IEC in the context of viral infections [22].

The aim of the present study was (i) to establish and culture chicken IEC in vitro, (ii) to monitor the infection of FAdV-4 strains, and (iii) to examine the pathogenicity of different strains in these cells. In this way, the initial host–pathogen interaction and signalling patterns of the immune response using different virulent isolates of the virus should be revealed.

## Materials and methods

### Isolation of chicken epithelial cells

**Figure 1:**
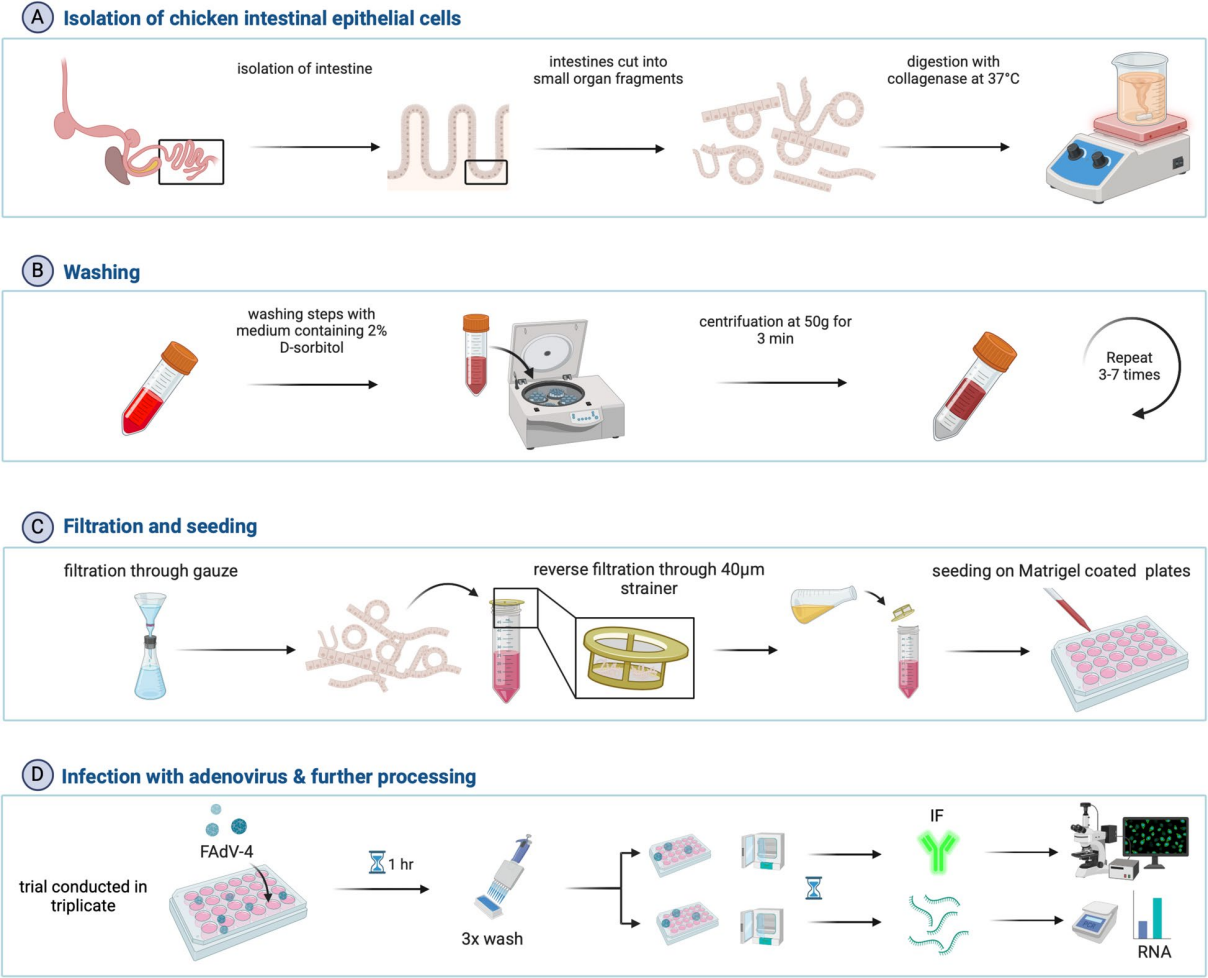
**Detailed scheme illustrating IEC culture steps, infection, and analyses.** **A** The process of IEC isolation from chicken small intestine, **B** 3–7 washing steps to ensure purity of the isolated cells, **C** filtration through gauze and a 40 µm strainer, followed by seeding of the IEC and **D** infection of the seeded IEC with FAdV-4, followed by subsequent processing; IF: immunofluorescence.

The experiment was conducted in triplicate using 240 embryonated specific pathogen-free eggs (VALO BioMedia GmbH, Osterholz-Scharmbeck, Germany) for each. The eggs were incubated at 37 °C, with 5% CO_2_ and 65% relative humidity, and were regularly turned to ensure optimum hatching conditions. After 14 days of incubation, the eggs were opened, and the embryos were killed by decapitation. For the isolation of IEC, the protocol of Kaiser et al. [23] was applied with slight modifications (Figure 1). Briefly, the entire intestine was removed and flushed with pre-warmed Hanks’ Balanced Salt Solution (HBSS) (Gibco™, Thermo Fisher Scientific, Waltham, USA). Subsequently, the organ was cut longitudinally into pieces of ~1 mm in length and washed thoroughly in 37 °C HBSS to remove intestinal content. The organ fragments were digested in a digestion medium (Table 1) for 50 min at 37 °C with continuous agitation (Figure 1A). After centrifugation (460 RS; Hettich Rotanta, Malmö, Sweden) at 100 × *g* for 3 min, the cell pellet was resuspended in a washing medium (Table 1) and centrifuged again at 50 × *g* for 3 min. The washing process was repeated until the supernatant became clear (Figure 1B). The remaining tissue particles were filtrated through sterile gauze, followed by centrifugation at 50 × *g* for 3 min and filtrated through a 40 µm nylon cell strainer (BD Falcon, BD Bioscience, San Jose, USA). The obtained cell aggregates were resuspended in 50 mL pre-warmed growth medium (Table 1) and counted using a Neubauer haemocytometer (VWR Scientifics, West Chester, USA).

**Table 1:** **Composition of used media**.

Medium	Composition
Digestion medium	• DMEM/F12 + Glutamax (Gibco™, Thermo Fisher Scientific) • 0.2% collagenase (Sigma-Aldrich, St. Louis, MO, USA)
Washing medium	• DMEM/F12 + Glutamax (Gibco™, Thermo Fisher Scientific) • 2% D-sorbitol (Carl Roth GmbH, Karlsruhe, Germany)
Growth medium	• DMEM/F12 Glutamax (Gibco™, Thermo Fisher Scientific) • 5% foetal calf serum (Gibco™, Thermo Fisher Scientific) • 100 U/mL penicillin–streptomycin (Thermo Fisher Scientific) • 20 ng/mL human epidermal growth factor (Thermo Fisher Scientific) • 2 µg/mL insulin (Sigma-Aldrich) • 1.4 µg/mL hydrocortisone (Sigma-Aldrich) • 1 µg/mL fibronectin (Sigma-Aldrich) • 5 units transferrin (Sigma-Aldrich) • 0.1 mg/mL heparin sodium salt (Sigma-Aldrich) • 10 mM HEPES (Gibco™, Thermo Fisher Scientific)
Maintenance medium	• DMEM/F12 Glutamax (Gibco™, Thermo Fisher Scientific) • 2% foetal calf serum (Gibco™, Thermo Fisher Scientific) • 20 ng/mL human epidermal growth factor (Sigma-Aldrich) • 2 µg/mL insulin (Sigma-Aldrich, St. Louis, MO, USA) • 100U/mL penicillin–streptomycin (Thermo Fisher Scientific) • 0.1 mg/mL heparin sodium salt (Sigma-Aldrich)

A 100 µL medium containing approximately 100 cell aggregates was seeded onto 24-well plates coated with Matrigel^®^ (Corning Incorporated, Corning, New York, USA) per well (Figure 1C). The plates were kept at 37 °C and 5% CO_2_ in the incubator (Heracell 150, Thermo Fisher Scientific). Small intestinal crypt-like structures and substantial cell monolayer outgrowth dissociation were observed during incubation within 24 h. The cells originated from collected intestinal crypts, with expansive zones of interconnected growth. Morphological analysis revealed an epithelial-specific cobblestone shape of outgrowing cells, which was previously described as characteristic of this cell type [24]. Unattached cell aggregates and unattached virus were removed by washing with pre-warmed HBSS (Figure 1D).

### Virus preparation

The FAdV-4 strain AG234 (GenBank accession number MK572849), previously shown to be highly virulent [11] and the non-pathogenic reference strain KR5 (GenBank accession number HE608152) were used. Both viruses underwent plaque purification thrice before being propagated on primary chicken-embryo liver (CEL) cells following the protocol of Schat and Sellers [25] to ensure purity. The virus titers for both viruses were determined to be 10^7^ tissue culture infectious dose 50 (TCID_50_)/mL, calculated through endpoint titration, as originally described by Reed and Muench [26]. The virus suspensions were kept at −40 °C until further use.

### Infection with FAdV-4 strains

The field strain AG234, and the reference strain KR5 were added to separate cell cultures in 24-well plates for further incubation, using 50 µL of virus suspension for each. The described experiment was conducted in triplicate, ensuring the reproducibility of the results (Figure 1D). Consequently, IEC in nine wells was infected with each virus strain for immunofluorescence analysis, while 6 wells served as NC (negative control) at each time point. Additionally, for reverse transcription-quantitative polymerase chain reaction (RT-qPCR) analysis, 8 wells per time point were used for infection, and 8 wells served as the NC. After incubation for one hour, the medium was replaced with a fresh maintenance medium (Table 1). After 4, 12, 24 and 48 hours post-infection (hpi), the cells underwent either fixation with ice-cold methanol for three minutes for immunofluorescence or immersion in the RNA stabilisation reagent RNA-later (Qiagen, Hilden, Germany), followed by freezing at −80 °C for further mRNA analysis.

### Image analysis

#### Immunofluorescence

The methanol-fixed cells were blocked with normal goat serum (Szabo-Scandic, Vienna, Austria) at room temperature (RT) for 1 h before being rinsed three times with PBS (Gibco^®^, Thermo Fisher Scientific) for 5 min. The samples were then incubated overnight at 4 °C with a polyclonal rabbit antibody, raised against strain KR5 in a dilution of 1:1000. The antibody production in rabbits has been described previously by De Luca et al. [27]. After three additional 5 min washing steps, the cells were incubated for 1 h at RT with E-cadherin mouse antibody (Code 7D6, dilution: 1:4.5; Developmental Studies Hybridoma Bank, Iowa City, IA, USA), a specific marker for chicken epithelial cells. Subsequently, the antibody was removed by rinsing the cells with PBS (pH 7.4) thrice for 5 min. The cells were then further incubated for 1 h with fluorescent-labelled secondary antibodies, anti-mouse Alexa Fluor 488 1:500 and anti-rabbit Alexa Fluor 657 1:1000 (both Invitrogen™, Thermo Fisher Scientific). Following further washing with PBS, nucleus staining was performed by adding DAPI (1:1000) for 5 min. The samples were then mounted in Aqua-Poly/Mount (Polysciences, Eppelheim, Germany).

#### Determination of epithelial cell growth and virus dissemination

**Figure 2:**
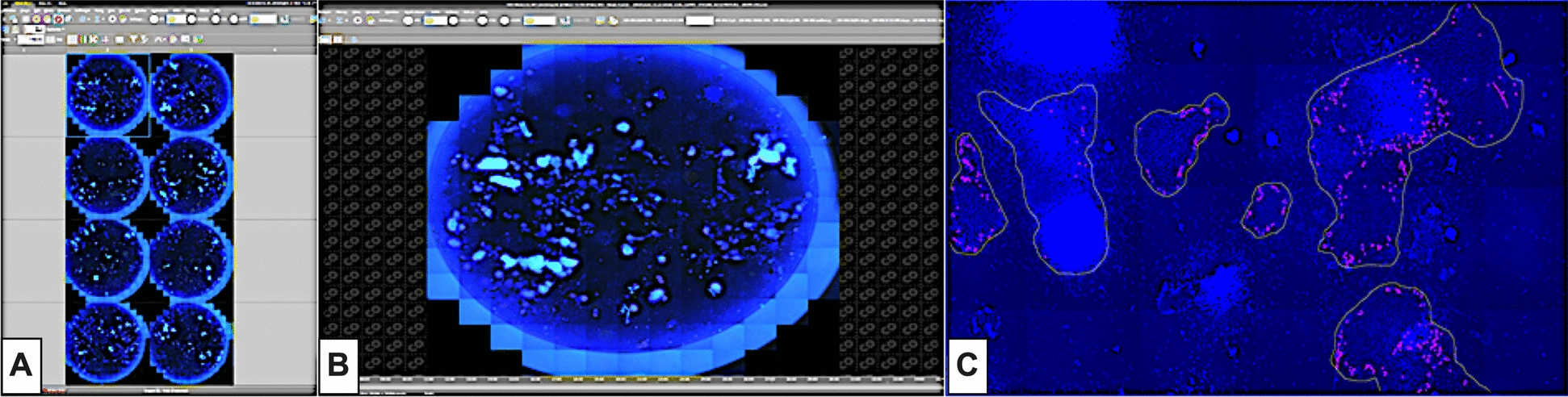
**Concept overview of the analysis of epithelial and/or infected cell areas utilising the open-source application “Fiji” at a magnification of 20x.** **A** Overview of wells that were measured. **B** Each well was selected to define the ROI. **C** Identification of virus-positive cells (labelled by a red mask) in an epithelial cell area.

An inverted spinning disk microscope (Nikon Eclipse Ti2-E/Yokogawa CSU-W1 Spinning Disk) was used for high-speed multidimensional imaging. Individual scans were conducted on 24-well plates at a magnification of 20x, utilising fluorescence channels to detect DAPI (channel Ex405/Em440 nm), FITC (channel Ex488/Em521 nm), and TRIC (channel Ex561/Em600 nm). Furthermore, a region of interest (ROI) was defined as encompassing almost the entire well, with a size of 122.51 mm^2^ (Figure 2). The scans were analysed using the open-source application Fiji [28], which required programming a specific macro. Briefly, the fluorescence channels were split to detect FITC-labelled epithelium, TRIC-labelled virus particles, and DAPI-stained nuclei separately. The fluorescence signals were detected according to an individual threshold intensity value, and the fraction of virus signals within the epithelium was calculated. Masks for nuclei (channel Ex405/Em440 nm) were created using the Auto Local Threshold plugin with Bernsen’s thresholding method (radius = 15 pixels).

### Gene expression analyses

For RT-qPCR, total RNA was extracted from the respective wells using the Qiagen RNeasy Mini Kit (Valencia, CA, USA) according to the manufacturer’s instructions. NanoDrop 2000 (Thermo Fisher Scientific) was used to analyse the A260/230 ratio to ensure the nucleic acid purity of the RNA obtained. Furthermore, Bioanalyzer 2100 chip-based capillary electrophoresis (Agilent Technologies, Waldbronn, Germany) was utilised to measure the RNA quality and quantity. RT-qPCR determined the gene expression of the cytokines IL-1β, IL-6, IL-10, IL-13, and IFN-γ, as well as TLR1B, 2B, 3, 4, and 21 [29–31]. The primers and probe sequences are shown in Additional file 1. For the normalisation of gene expression, the reference genes RPL13 (Ribosomal Protein L13) and TBP (TATA box binding protein) were targeted using previously published primers and probes [32] (Additional file 1).

To transcribe cytokine and TLR mRNA into cDNA and amplify the latter, we applied the Brilliant III Ultra-Fast RT-qPCR Master Mix kit (Agilent Technologies) in combination with the AriaMx real-time PCR system (Agilent Technologies) and the Agilent AriaMx1.0 software (Agilent Technologies). The Ct values of cytokines and TLRs were normalised using the Ct values of the reference genes. The thermal cycle profile for RT-qPCR was as follows: 1 cycle of reverse transcription at 50 °C for 10 min and 95 °C for 3 min to initiate hot start, followed by 40 cycles of amplification at 95 °C for 5 s and 60 °C for 10 s.

All samples were analysed with various controls, such as NRT (non-reverse transcriptase) and NTC (non-template control), to determine any possible genomic DNA contamination. Fold changes were calculated using the 2^−∆∆CT^ formula to assess the results. This formula was required to set the ∆CT of each group, which was determined by subtracting the CT or the target gene from the CT of the reference gene. A control average was defined to enable accurate measurement and statistical analysis of the fold change between the NC and the infected IEC. The latter was calculated by taking the mean of the NC ∆CT values. Subsequently, the ∆∆CT value was obtained by subtracting the control average from the ∆CT value of the infected sample. The ∆∆CT value was further used to obtain the final 2^−∆∆CT^, which yielded the fold change values.

### Statistical analysis

Before further analysis, data for epithelial area, infected area, and gene expression were screened for outliers using ROUT outlier statistics (Q = 1%). The normality of data distribution was assessed using the Shapiro–Wilk test. The observed non-normal distribution in a subset of the data measuring epithelial and infected areas suggested that a non-parametric approach was appropriate. Accordingly, the Kruskal–Wallis Test was used for comparative analysis across numerous groups. Multiple comparisons were calculated by controlling the false discovery rate (FDR) using the two-stage linear step-up procedure developed by Benjamini et al. [33].

For the gene expression analysis, a log_10_ transformation was applied to align the data with the assumption of a normal distribution, thereby enabling parametric statistics. Employing a two-way analysis of variance (ANOVA), we compared gene expression levels between strains AG234 and KR5 against the uninfected control across time points at 4, 12, 24, and 48 hpi. Following ANOVA, Dunn’s multiple comparisons test was used for multiple group comparisons. *P*-values less than 0.05 were considered significant. All statistical analyses were performed using GraphPad Prism version 9 (GraphPad Software, CA, USA).

## Results

### Image analysis

#### Determination of epithelial cell area after FAdV-4 infection

**Figure 3:**
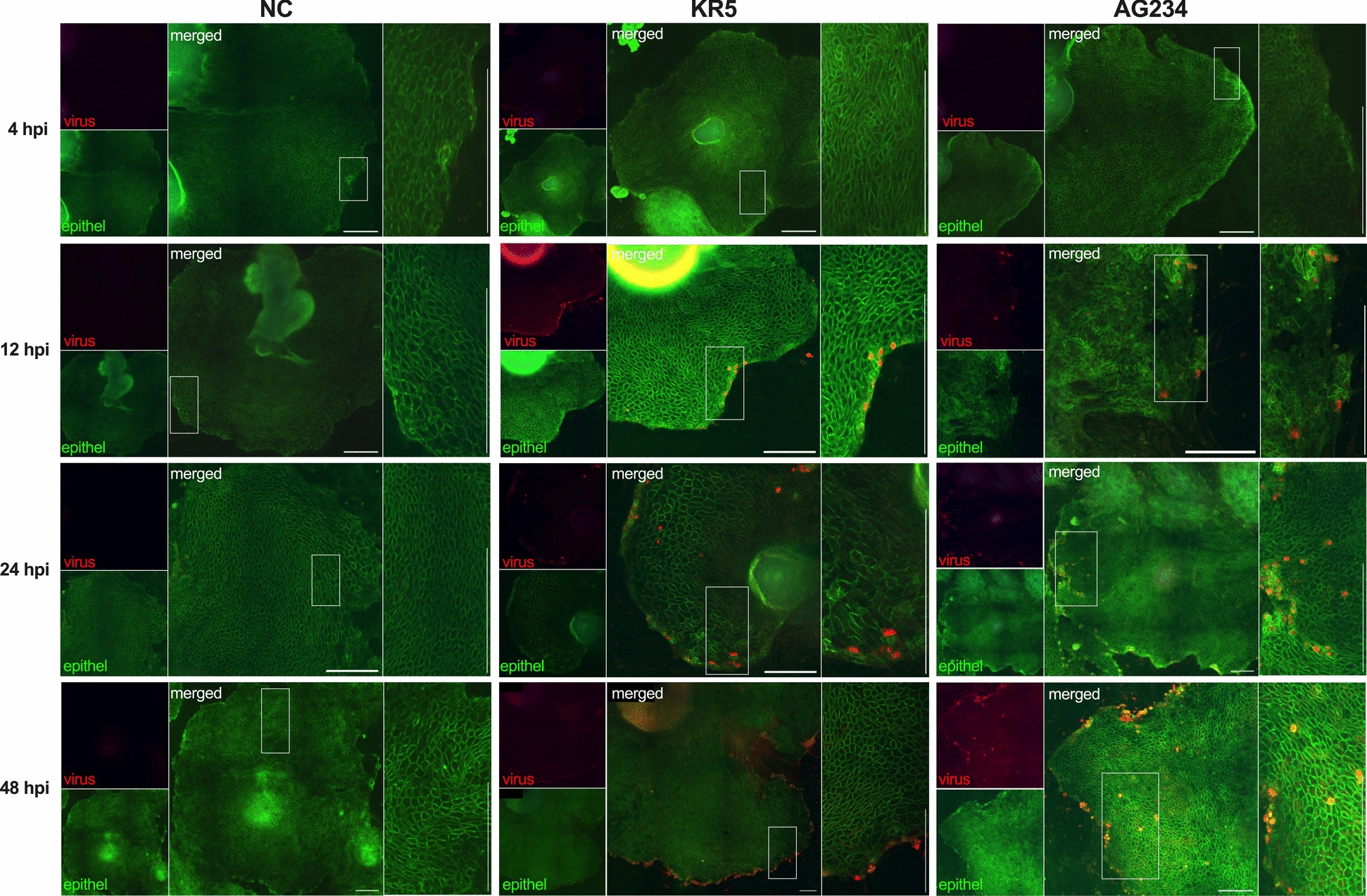
**Representative illustration depicting the temporal changes of non-infected and virus-positive cells at 4, 12, 24 and 48 hpi.** Virus-positive cells, either KR5 or AG234, are co-stained in red (Alexa Fluor 657), while non-infected epithelial cells are stained green (Alexa Fluor 488) only. The illustration demonstrates a time-dependent increase in the area of infected epithelial cells, reaching its peak at 48 hpi. The negative control (NC) remained negative throughout the experiment. Boxed regions mark representative details in higher magnification (right side of each picture). Scale bars indicate 250 µm.

**Figure 4:**
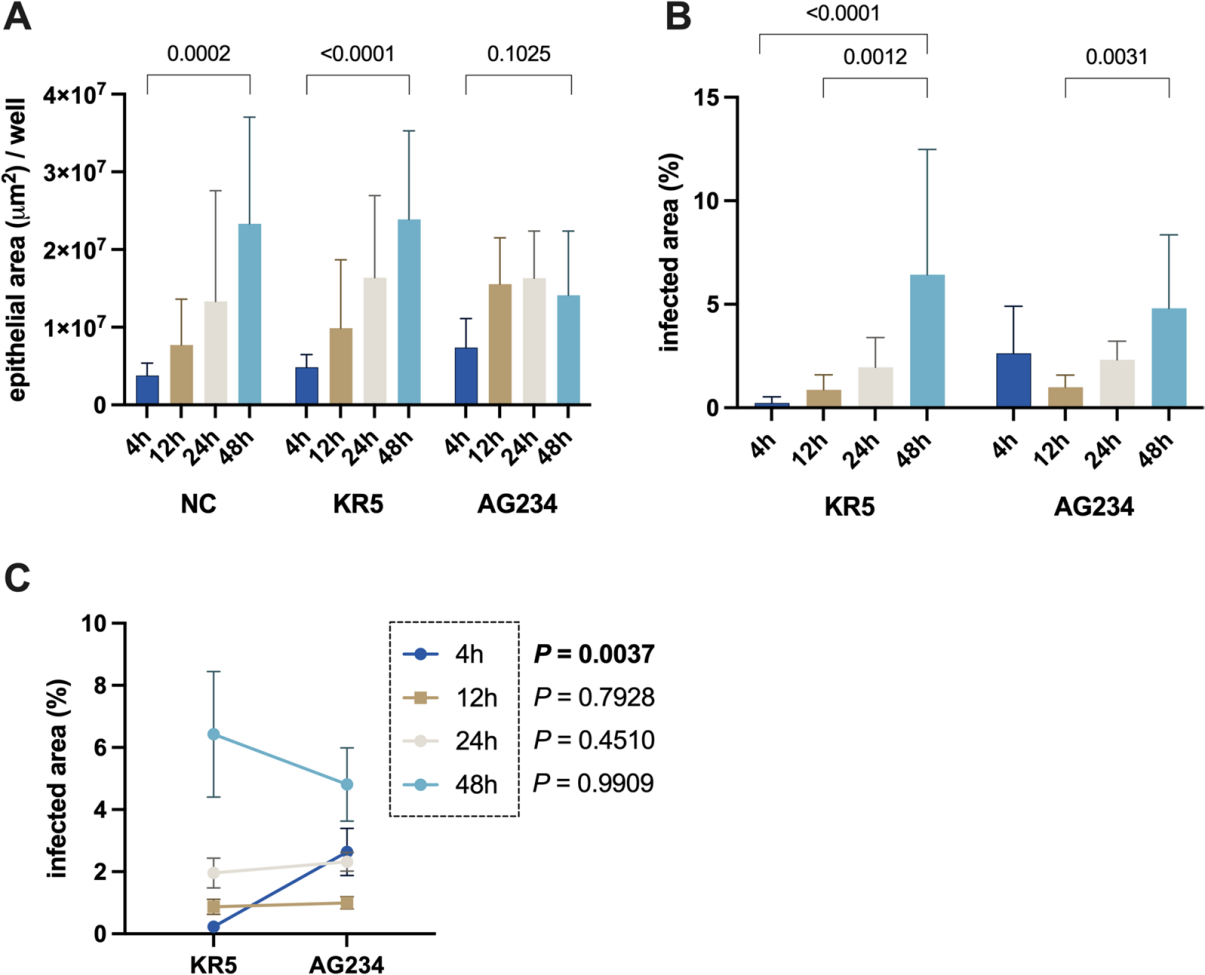
**Primary IEC following infection with KR5, AG234, or without virus (NC).** **A** Comparison of the mean ± SEM for IEC areas with statistical *P*-values calculated within the values of NC, KR5 and AG234 at time points 4, 12, 24 and 48 hpi. **B** Mean percentages of virus-positive cell areas ± SEM within the IEC areas, with statistical differences calculated between time points within KR5 and AG234 infected cultures. **C** Comparison of mean ± SEM for AG234 and KR5 of the infected area between KR5 and AG234. Data presented as mean ± SD. Significance indicated by *P* < 0.05.

To investigate changes in IEC growth following FAdV-4 infection, we measured the total cell-covered area and virus-infected cell area within the ROI. Immunofluorescence indicated the presence of both virus strains, KR5 and AG234, in the IEC up to 48 hpi (Figure 3). At 4 hpi, the mean uninfected IEC areas measured 3.77 × 10^6^ µm^2^, increasing to 7.73 × 10^6^ µm^2^ at 12 hpi, 1.33 × 10^7^ µm^2^ at 24 hpi and reaching 2.33 × 10^7^ µm^2^ at 48 hpi. Notably, a significant difference was observed between 4 and 48 hpi (Figure 4A).

In KR5-infected cells, the epithelial cell area increased over time, mirroring the trend observed in the NC group. At 4 hpi, the mean cell area was measured at 4.86 × 10^6^ µm^2^, rising progressively to 9.90 × 10^6^ µm^2^ at 12 hpi, 1.64 × 10^7^ µm^2^ at 24 hpi and peaking at 2.39 × 10^7^ µm^2^ at 48 hpi (Figure 4A). A significant expansion in the size of the epithelial area was evident in KR5-infected cells over the course of 4 to 48 hpi, indicating the continuous proliferation of cells. At all time points, epithelial cell areas infected with KR5 were slightly larger than in NC, but there was no significant difference.

In contrast, the culture infected with AG234 demonstrated a different growth pattern. The initial cell area at 4 hpi was larger than that of KR5 cells, recorded at 7.40 × 10^6^ µm^2^. Over time, the cell area fluctuated, reaching 1.55 × 10^7^ µm^2^ at 12 hpi, remaining stable at 1.63 × 10^7^ µm^2^ at 24 hpi and declining to 1.41 × 10^7^ µm^2^ at 48 hpi, thus falling below the level observed in the NC group (Figure 4A). However, the variations did not reach statistical significance. Overall, AG234 induced a non-significant increase in epithelial cell growth between 4 and 48 hpi.

#### Determination of virus-positive cell areas

At 4 hpi, KR5 exhibited an initial virus-infected cell area of 0.67%, while AG234 showed a significantly higher proportion of 2.64% (Figures 4B and C). By 12 hpi, the proportion for KR5 increased slightly to 0.87%, whereas AG234 decreased to 0.97%. At 24 hpi, KR5 reached 1.96%, slightly below AG234 at 2.32%. However, by 48 hpi, KR5 displayed a markedly higher percentage of 6.43% compared to 4.81% for AG234. Notably, the KR5 virus-positive cells exhibited significant variations between 4 and 48 hpi and between 12 and 48 hpi. In contrast, AG234-infected cells demonstrated a unique significant increase at 48 hpi compared to 12 hpi (Figure 4B). No area of virus-positive cells was determined in the NC group.

### Gene expression analyses

#### Toll-like receptors

**Figure 5:**
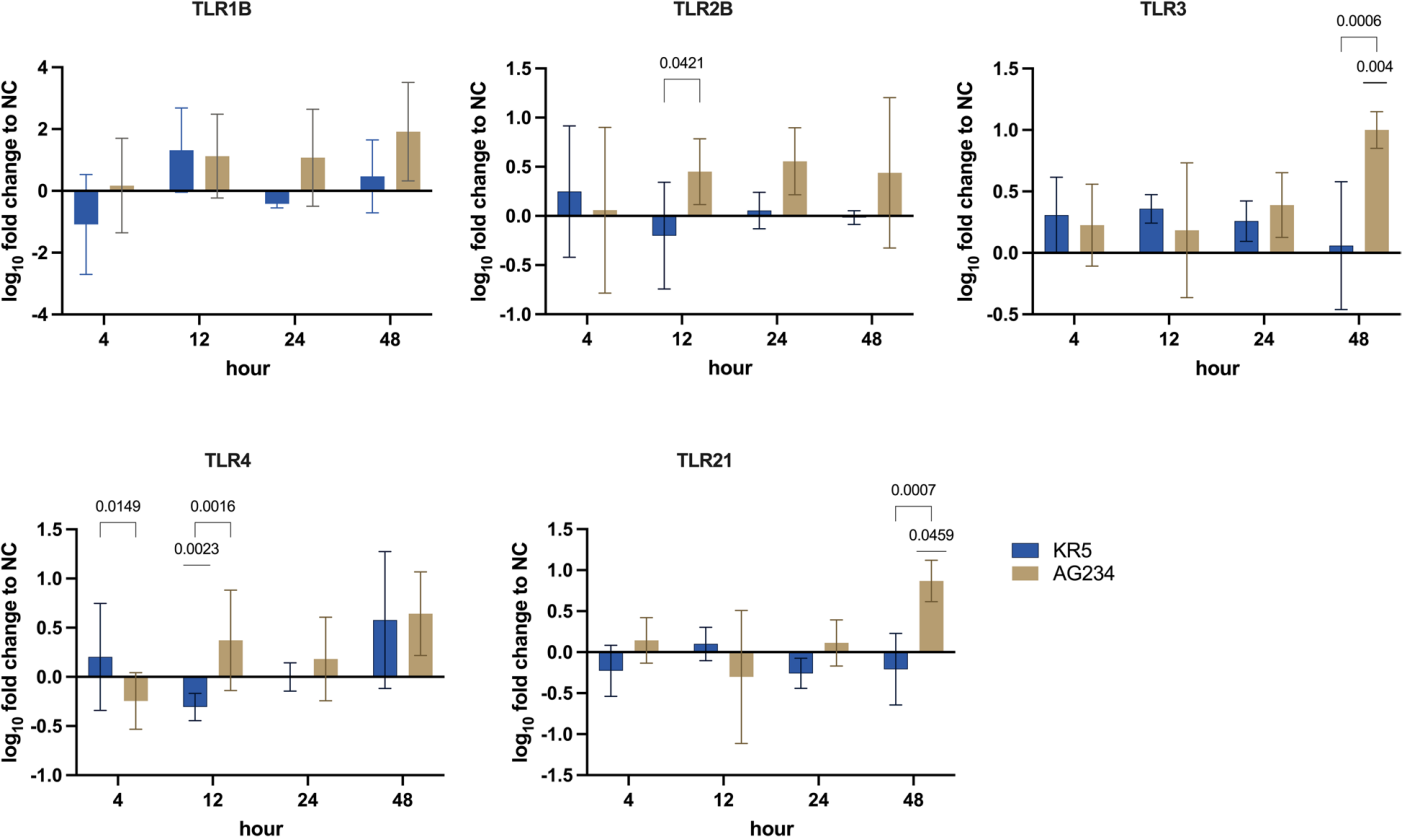
**Expression pattern of TLR 1B, 2B, 3, 4 and 21 in intestinal epithelial cell cultures at 4, 12, 24, 48 hpi following infection with KR5 and AG234 indicated by fold changes to the NC.** Significant differences were calculated for infected groups compared to the respective control group and between infected groups. Data presented as mean ± SD. Significant changes are indicated in case *P* < 0.05.

AG234 caused significantly higher expressions of several TLRs at certain time points compared to KR5 (Figure 5). Notably, at 4 hpi, TLR4 expression was significantly higher in KR5 than in AG234. At 12 hpi, both TLR2B and TLR4 expression levels were significantly higher in AG234 than in KR5. Additionally, TLR4 expression in KR5 was significantly lower than in the NC group at this time point. At the final time point (48 hpi), TLR3 and TLR21 expression levels were considerably higher in AG234 than in the NC and KR5.

#### Cytokines

**Figure 6:**
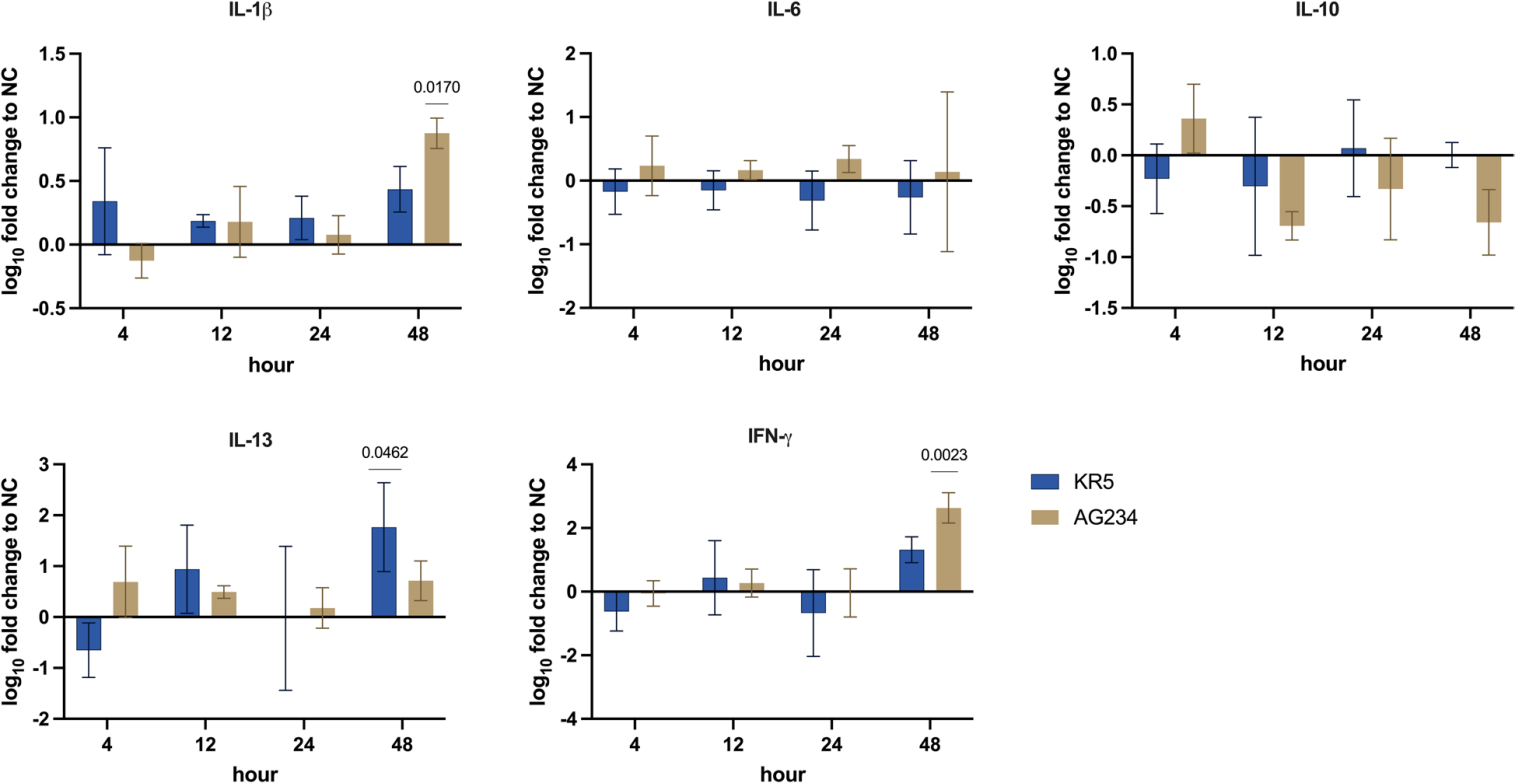
**Gene expression levels of IL-1β, IL-6, IL-10, IL-13 and IFN-γ in intestinal epithelial cell culture at 4, 12, 24, 48 hpi following infection with KR5 and AG234 indicated by fold changes to the NC.** Significant differences were calculated for infected groups compared to the respective control group and between infected groups. Data presented as mean ± SD. Significant changes are indicated in case *P* < 0.05.

At 4, 12, and 24 hpi, no significant differences in cytokine expressions were observed between the two strains or in comparison to the NC (Figure 6). However, at 48 hpi, IL-1β and IFN-γ expression levels were significantly higher following infection with AG234 than the NC. At the same time, IL-13 expression was significantly increased compared to the NC after infection with KR5.

## Discussion

The present study established an in vitro infection model for FAdV-4 using chicken-embryo epithelial cell culture. The study’s objectives were: i) to culture chicken IEC in vitro, ii) to monitor infection dynamics of FAdV-4 strains, and iii) to compare different pathogenicity in these cells. To investigate cell proliferation and viral dissemination in culture, we employed digital image analysis from 4 to 48 hpi. The open-source software ImageJ (Fiji) was used to analyse and visualise digital scans of cellular growth patterns based on applied markers, including E-Cadherin, a widely used marker for assessing cell–cell adhesion and epithelial integrity [34].

E-Cadherin expression provides insights into cellular viability, suggesting strong cell–cell adhesion, while disruptions indicate compromised adhesion, increased cell detachment, and potential cell stress or apoptosis [35]. Our aim was to detect changes in E-Cadherin expression to assess cell stress or death associated with FAdV strains of differing virulence. The imaging software used has previously been applied to quantify intestinal stem cell markers, asses epithelial monolayer formation [36] and measure cell numbers and tissue coverage in 2D versus 3D cultures in mice [37]. It ensures reproducibility through a consistent analysis pipeline, automates repetitive tasks, and quantifies parameters such as cell area, intensity, and shape descriptors [28]. This method proved effective for analysing tissue coverage and viral signals after FAdV-4 infection.

In this study, the epithelial area of cells infected with virulent or non-pathogenic FAdV strains was larger than that of the NC during the early phase of infection at 4 hpi. Although this effect was not statistically significant, one possible explanation is that adenoviruses create optimal conditions for replication by inducing infected host cells to enter the replicative S-phase [38, 39]. This manipulation promotes viral replication and modulates the immune response. Mammalian adenoviruses, for instance, can exert both synergistic and antagonistic effects on immunity [40]. Synergistically, human adenoviruses have been shown to function as adjuvants, enhancing the immune response to other antigens, a property utilised in vaccine development to amplify immune responses [41].

FAdVs remain less well-characterised than human adenoviruses, particularly regarding their roles in immune evasion and pathogenesis. Recent findings suggest that host proteins such as heat shock protein 70 (Hsp70) and its cofactor, DnaJ heat shock protein 40 family member C7 (DnaJC7), exert a synergistic effect in regulating FAdV-4 replication by interacting with the viral hexon protein, promoting autophagic degradation and inhibiting infection [42]. Human adenoviruses are also known to employ antagonistic immune evasion strategies, including inhibiting apoptosis and interferon activity. These strategies enable replication while avoiding premature cell death [43]. This alignment is consistent with our findings, as the larger epithelial area observed during the early infection phase suggests effective viral replication and persistence, likely supported by such immune evasion mechanisms.

After 12 hpi, cell growth continued in all groups, persisting until 48 h in KR5-infected cells and the NC group. In contrast, cell growth declined below NC levels after 24 hpi in IEC infected with the virulent strain AG234. At this stage, the pathogen is believed to control host cells fully, shifting its strategy from reproduction to efficient release and spread through cell destruction [44]. Our findings are, therefore, consistent with previous reports documenting early viral infection of cells and a peak in virus signal at 48 hpi in IEC. Although viral signals were initially much higher in AG234 than in those infected with KR5, this trend reversed over time, correlating with the stronger inhibition of cell growth by AG234.

To gain detailed insights into the innate immune response, the expression of host cytokines and TLRs was comparatively analysed at multiple time points. Significant responses were observed in TLR2B, 3, 4, and 21, as well as in IL-1β, IL-13, and IFN-γ. TLR2 binds to various ligands on microorganisms, including bacteria, fungi, viruses, and parasites [30]. In our study, AG234-infected cells exhibited significantly higher expression levels of TLR2B at 12 hpi compared to KR5, although not in comparison to the NC group. This difference was primarily attributable to the down-regulation of TLR2B in the KR5-infected cells. Supporting our observations, previous research has highlighted an increase in TLR2B expression in chickens following FAdV infection, particularly in the spleen, liver, bursa of Fabricius and LMH cells, indicating the initial signs of a potent innate immune response triggered by FAdV-4 infection in the host [14, 45].

The down-regulation of TLR2B in KR5-infected cells suggests a complex interaction with the host immune response and indicates differences in immune modulation compared to AG234. However, the mechanisms underlying reduced TLR2 B expression in the context of an FAdV-4 infection warrant further investigations. Viral replication generates double-stranded RNA (dsRNA), activating type I and II interferons via TLR3 recognition in diverse cell types, including IEC [46]. In our study, TLR3 expression was significantly elevated in IEC at 48 hpi. The interpretation of this outcome will be conducted collectively along with those for TLR21 and IFN-γ below.

Notably, significant differences in TLR4 expression between the strains were observed as early as 4 hpi and 12 hpi, suggesting an early and potentially crucial role for TLR4 in the prompt defence against viral intrusion. However, TLR4 is primarily known for recognising bacterial lipopolysaccharide (LPS) [30]. The marked down-regulation of TLR4 in KR5-infected cells compared to the NC group at 12 hpi may reflect a compensatory response to its earlier upregulation at 4 hpi, a pattern not observed in AG234-infected cells. He et al. (2007) [47] previously reported that TLR3 and TLR21 synergistically induce the expression of Th1 cytokine IFN-γ in response to FAdV-4 infection, as the TLR21 ligand CpG ODN directly stimulates innate immune cells. Our findings are consistent with these results, showing a significant increase in TLR3, TLR21, and IFN-γ expression at 48 hpi, specifically in cells infected with the virulent strain AG234. This finding suggests the activation of antiviral defence mechanisms by epithelial cells in response to AG234.

The two viral strains exhibited distinct cytokine expression profiles, with significantly higher levels of the pro-inflammatory cytokine IL-1β observed in AG234-infected cells at 48 hpi. IL-1β signalling triggers an interferon-like response, activating interferon-stimulated genes that are key in restricting viral replication. This antiviral response is mediated by an IRF1-dependent pathway essential for inducing antiviral genes downstream of the IL-1 receptor [48]. However, excessive IL-1β can lead to tissue damage and may facilitate viral spread by disrupting epithelial barriers [49]. A previous study documented a significant increase in IL-1β expression in chicken kidney cells infected with FAdV-4, observed at 3 dpi [50]. Our findings suggest that the elevated viral signals observed in the image data may intensify and prolong IL-1β induction, with AG234-infected cells exhibiting a particularly strong response at 48 hpi. This pronounced IL-1β induction likely enhances the activation of the IRF1-dependent pathway, resulting in increased expression of antiviral genes downstream of the IL-1 receptor.

Consistent with this, our study showed a reduction in the epithelial area after 48 hpi in cells infected with the virulent AG234 strain, compared to KR5 and the negative control. This outcome may indicate tissue damage due to excessive IL-1β expression. Meanwhile, IL-6 and IL-10 levels were not significantly altered, which could be attributed to the cell type. In contrast, previous studies have reported significantly increased IL-6 expression across multiple organs in chickens infected with virulent FAdV-4, most likely by immune cells [44]. IL-13, originating from monocytes or macrophages, exerts an anti-inflammatory effect by down-regulating the production of pro-inflammatory mediators, including prostaglandins, reactive oxygen, and nitrogen species [51]. The production suppression of these pro-inflammatory cytokines helps prevent tissue damage during viral infections [52].

In our study, this interleukin was notably upregulated at 48 hpi with the attenuated virus strain KR5 compared to the AG234 and NC strains. Increased IL-13 expression may help to mitigate inflammation and support tissue repair, whereas the higher replication rate of the AG234 strain could trigger a stronger inflammatory response, resulting in greater tissue damage. The finding also suggests that modulation of an inflammatory response may be linked to the down-regulation of TLR4 expression observed in KR5-infected cells, indicating a potential mechanism by which the attenuated virus strain exerts immunomodulatory effects [3, 53]. Furthermore, IL-13 is key in promoting Th2-type immune responses [54]. By inducing IL-13, KR5 may shift the immune response towards a Th2 activation, which could suppress Th1-driven antiviral immunity, including cytotoxic T cell responses and interferon production. IL-13 has also been shown to promote the differentiation of regulatory T cells (Tregs), which are critical for controlling immune responses and maintaining immune homeostasis [55]. Therefore, IL-13 induction by KR5 could enhance Treg responses, further suppressing immune activation and limiting the host’s ability to mount an effective antiviral response.

Genetic differences within the genome and their interaction with the host immune response may influence the virulence in different FAdV strains. A previous study reported that a single amino acid substitution in the hexon protein can determine virulence, a finding also confirmed for the strains used in the actual study [10]. The observed differences in immune response may be further attributed to the hexon protein’s critical role in FAdV-4’s ability to evade the host immune system during antigen recognition, resulting in viral persistence and increasing virulence [3]. Additionally, variations in the fibre-1 and fibre-2 proteins could influence viral replication [56]. Additionally, fibre protein, particularly in recombinant forms, has been shown to stimulate robust local cellular immune responses, which enhance protection against diseases like HHS [53]. However, FAdV-4 can manipulate these pathways to favour its replication and persistence [57]. In our study, the virulent strain was associated with reduced epithelial growth, possibly due to the down-regulation of signalling pathways involved in tissue repair and regeneration. This effect may contribute to the severe pathological manifestations observed in infected tissues, warranting further investigation into the underlying molecular mechanisms.

Although AG234 induces the up-regulation of TLR3, TLR21, IL-1β, and IFN-γ at 48 hpi, the virus might manipulate the timing of this response to prevent overactivation of immune pathways that could otherwise lead to a more effective viral clearance. Altering the balance of cytokine production could delay or limit immune cell activation at critical points, allowing the virus to persist longer before the immune response becomes fully activated [58]. Additionally, AG234 may suppress downstream signalling required to activate immune cells such as dendritic cells, macrophages, or T cells, enabling selective immune modulation and evasion of complete immune detection while still triggering inflammation. Despite the up-regulation of IFN-γ, AG234 may interfere with the antiviral effects of other interferons or their respective ligands by disrupting the JAK-STAT signalling pathway or encoding viral proteins that inhibit interferon responses. These strategies would dampen antiviral defence mechanisms, as described previously [59]. Further investigations are needed to explore these potential immune evasion mechanisms in more detail.

Overall, it is important to note that the intestine is populated by diverse immune cells in vivo, which play essential roles in orchestrating immune responses against pathogens. Therefore, further studies incorporating immune cell populations are needed to fully understand the complexity of the intestinal immune response to FAdV-4 infection. With the in vitro model now established, there are opportunities to broaden the analysis by including additional immune markers, particularly through transcriptomic approaches. Moreover, the mRNA expression data obtained in this study can serve as a cornerstone for protein-detection methods, such as western blot or ELISA, enabling deeper insights into virus-host interactions. These investigations will contribute to a more refined understanding of FAdV infection and immune modulation on the protein level.

Despite its limitations, this study provides valuable new insights into the differential impacts of two FAdV-4 strains on cell growth and the innate immune response according to their virulence. Furthermore, the established primary cell culture system offers a useful platform for studying interactions between various intestinal pathogens and host cells, potentially facilitating the development of early intervention strategies.

## Supplementary Information

**Additional file 1: RT-qPCR primers and probes for gene expression of Toll-like receptors, interleukinsand interferon gamma.** Ribosomal protein L13and TATA-binding proteinwere applied as reference genes.

## Data Availability

The datasets supporting the conclusions of this article are included within the article and its additional files.
