# Supplementary material for: Virulence of fowl adenovirus (FAdV) serotype 4 strains impacts cell proliferation and immune response of primary chicken-embryo intestinal epithelial cells
Source: Vet Res. 2025 May 27;56:109. doi: 10.1186/s13567-025-01541-9 (PMC12107922; doi:10.1186/s13567-025-01541-9)
Supplement: Supplementary file 1 — Additional file 1: RT-qPCR primers and probes for gene expression of Toll-like receptors, interleukinsand interferon gamma. Ribosomal protein L13and TATA-binding proteinwere applied as reference genes. [file 13567_2025_1541_MOESM1_ESM.docx]

**Additional file 1 RT-qPCR primers and probes for gene expression of Toll-like receptors (TLR), interleukins (IL) and interferon gamma (INF-γ).** Ribosomal protein L13 (RPL13) and TATA-binding protein (TBP) were applied as reference genes.

| **Gene** | **Used primer concentration (nM)** | **NCBI Accession No.** | **Primer and Probe Sequences (5‘-3‘)** |
| --- | --- | --- | --- |
| *TLR1B* | 400 | DQ518918.1 | F: CCATCACAAGTTGTTTAGC  R: TCCAGGTAGGTTCTCTTG  P: HEX-CCTGATCTTGCTGGAGCCGA-BHQ1 |
| *TLR2B* | 400 | AB046533.2 | F: GATCCCCAAGAGGTTCTG  R: CTGCTGTTGCTCTTCATC  P: FAM-CTGCGGAAGATAATGAACACCAAGAC-BHQ1 |
| *TLR3* | 400 | EF137861.1 | F: GCATAAGAAGGAGCAGGAAGA R: GGAGTCTCGACTTTGCTCAATA P: ROX-TGGTGCAGGAGGTTTAAGGTGCAT-BHQ2 |
| *TLR4* | 400 | KF697090.1 | F: CATACAAGCCACTCCAAGCC  R: AGGATTTCCAGGGCTGAGTC P: CY5-CACAGCTCTGGATTTCAGCAACAACCA-BBQ |
| *TLR21* | 400 | NM_001030558.1 | F: TCGCAACTGCATTGAGGATG R: ATGACAGATTGAGCGCGATG P: CY5-TTCCTGCAGTCGCCGGCCCT-BHQ2 |
| *IL-1β* | 400 | XM_046931582.1 | F: GCTCTACATGTCGTGTGTGATGAG  R: TGTCGATGTCCCGCATGA  P: CY5-CCACACTGCAGCTGGAGGAAGCC |
| *IL-6* | 600 | NM_204628.2 | F: GCTCGCCGGCTTCGA  R: GGTAGGTCTGAAAGGCGAACAG  P: HEX-AGGAGAAATGCCTGACGAAGCTCTCCA |
| *IL-10* | 400 | NM_001004414.4 | F: CATGCTGCTGGGCCTGAA  R: CGTCTCCTTGATCTGCTTGATG  P: ROX-CGACGATGCGGCGCTGTCA |
| *IL-13* | 600 | NM_001007085 | F: CACCCAGGGCATCCAGAA R: TCCGATCCTTGAAAGCCACTT P: CY5-CATTGCAAGGGACCTGCACTCCTCTG-BHQ1 |
| *INF-γ* | 600 | NM_205149.1 | F: GTGAAGAAGGTGAAAGATATCATGGA  R: GCTTTGCGCTGGATTCTCA  P: HEX-TGGCCAAGCTCCCGATGAACGA |
| *RPL13* | 500 | NM_204999.2 | F: GGAGGAGAAGAACTTCAAGGC  R: CCAAAGAGACGAGCGTTTG P: HEX-CTTTGCCAGCCTGCGCATG-BHQ1 |
| *TBP* | 400 | NM_205256.2 | F: CATTCCAGGTGCGTGAACTC  R: TTAAGCCTGGTGCTGGATCA  P: ROX-TCCTCGTCCTCCGCCGCGAG |
